# Reconsidering the Clinical Utility of a Body Shape Index for Abdominal Aortic Calcification in Hypertension: From Association to Clinical Applicability

**DOI:** 10.1002/clc.70372

**Published:** 2026-06-08

**Authors:** Dengfeng Zhang, Xiaoling Shang

**Affiliations:** ^1^ Changchun University of Chinese Medicine Changchun Jilin China

To the Editor,

We read with interest the recent article by Qiu and Wang, which investigated the association between A Body Shape Index (ABSI) and abdominal aortic calcification (AAC) in individuals with hypertension [1]. The authors should be commended for addressing an important clinical question and for their rigorous use of restricted cubic splines and subgroup analyses. However, several methodological and interpretative aspects warrant careful consideration before concluding that ABSI provides meaningful clinical value for risk stratification in this population.

First, while the authors report a statistically significant independent association between ABSI and AAC (OR per SD = 1.162, 95% CI: 1.025–1.318, *p* = 0.019), the effect size is modest, and the area under the ROC curve (AUC = 0.625) indicates only marginal discriminative ability. An AUC below 0.70 is generally considered insufficient for clinical decision‐making [2]. The authors themselves acknowledge that ABSI demonstrates “limited predictive ability” and should serve only as a “supplementary risk indicator.” This raises the fundamental question: does a statistically significant association with a modest effect size, in the absence of incremental predictive value over established risk factors, justify clinical attention? In biomarker evaluation, statistical significance should not be conflated with clinical utility [3].

Second, the cross‐sectional design precludes any causal inference. The authors observe that AAC and ABSI are measured simultaneously, making it impossible to determine whether elevated ABSI precedes calcification or whether both are consequences of shared pathophysiological processes (e.g., chronic inflammation, metabolic dysregulation). Moreover, ABSI—a composite of waist circumference, height, and weight—is inherently a static anthropometric index. However, body composition and fat distribution change over time, and a single baseline measurement cannot capture cumulative exposure. Previous studies have demonstrated that longitudinal trajectories of adiposity indices provide stronger prognostic information than single assessments [4]. The absence of repeated measurements in this study limits its relevance for long‐term risk prediction.

Third, the biological non‐specificity of ABSI deserves emphasis. ABSI has been associated with a wide range of cardiovascular outcomes, including arterial stiffness, coronary artery disease, and mortality, in both hypertensive and non‐hypertensive populations [5, 6]. The fact that ABSI predicts AAC in hypertensive patients may simply reflect its ability to capture generalized visceral adiposity and systemic inflammation—factors that are associated with virtually all forms of vascular disease. This lack of specificity is further supported by evidence from the general population, where ABSI has also been shown to be associated with AAC [7]. Thus, ABSI is unlikely to provide disease‐ or site‐specific information about AAC beyond what is already captured by conventional metabolic and inflammatory markers. This lack of specificity limits its incremental value in clinical practice.

Finally, the extensive subgroup analyses, while informative, were exploratory and not adjusted for multiple comparisons, increasing the risk of type I error. The authors appropriately caution readers about this limitation. However, the presentation of numerous statistically significant subgroup findings (e.g., in females, older age, non‐smokers, etc.) may inadvertently encourage overinterpretation. In the absence of a prespecified hypothesis and formal correction for multiplicity, these findings should be regarded as hypothesis‐generating rather than confirmatory.

Taken together, the study by Qiu and Wang provides valuable epidemiological evidence of an association between ABSI and AAC in hypertensive patients. However, the modest effect size, cross‐sectional design, biological non‐specificity, and lack of demonstrated incremental predictive value over established risk factors suggest that ABSI is unlikely to serve as a clinically actionable tool for AAC screening. Future prospective studies with serial ABSI measurements, head‐to‐head comparisons with existing risk scores, and hard clinical outcomes are needed to determine whether ABSI truly adds value beyond conventional risk assessment. Until then, ABSI should remain an exploratory research metric.

## Author Contributions

All authors contributed equally to the conception and writing of this manuscript.

## Funding

The authors have nothing to report.

## Ethics Statement

The authors have nothing to report.

## Conflicts of Interest

The authors declare no conflicts of interest.

## Data Availability

Data sharing is not applicable to this article as no data sets were generated or analyzed during the current study.
